# Physiological Correlates of Moral Decision-Making in the Professional Domain

**DOI:** 10.3390/brainsci9090229

**Published:** 2019-09-11

**Authors:** Michela Balconi, Giulia Fronda

**Affiliations:** 1 Department of Psychology, Catholic University of the Sacred Heart, 20123 Milan, Italy; michela.balconi@unicatt.it; 2 Research Unit in Affective and Social Neuroscience, Catholic University of the Sacred Heart, 20123 Milan, Italy

**Keywords:** moral decision-making, skin conductance response (SCR), heart rate (HR), heart rate variability (HRV), fairness, company

## Abstract

Moral decision-making is central to guide our social behavior, and it is based on emotional and cognitive reasoning processes. In the present research, we investigated the moral decision-making in a company context by the recording of autonomic responses (skin conductance response, heart rate frequency, and variability), in three different moral conditions (professional fit, company fit, social fit) and three different offers (fair, unfair, neutral). In particular, the first professional fit condition required participants to accept or reject some offers proposing the money subdivision for a work done together with a colleague. The second company fit condition required participants to evaluate offers regarding the investment of a part of the money in the introduction of some company’s benefits. Finally, the third social fit condition required participants to accept or refuse a money subdivision to support a colleague’s relative with health problems financially. Results underlined the significant effect of both the condition, with increased autonomic effects more for personal and social than company fit, and the offer type, with differences for fair and neutral offers compared to unfair ones. This research shows how individual, situational, and contextual factors influence moral decision-making in a company context.

## 1. Introduction

Moral decision-making represents a complex process that requires individuals to make consistent decisions in actions that can harm or help others, demanding a balanced achievement between personal and other interests, immediate or deferred rewards, and emotional and rational processes [1]. Some recent studies have shown that moral decision-making is mediated by two main computational processes [2,3]. The first concerns the moral intuition, consisting of an emotional process that allows individuals to evaluate socially relevant stimuli as right or wrong; the second concerns moral reasoning, consisting of some controlled deductive reasoning processes and cost-benefit analyses about potential outcomes of moral decisions [2,4]. Moreover, moral decision-making can be led by a deontological principle if the morality action evaluation depends on its intrinsic nature, or by a utilitarianism principle if the evaluation is based on action consequences [5].

To investigate the processes underlying moral decision-making, classic paradigms required individuals to decide by evaluating it according to their criteria and consisted mainly of monetary choices or mathematical exercises [6,7]. Moreover, other paradigms of social decision tasks have been used for the altruistic behavior and equity perception evaluation, such as the Ultimatum Game (UG). Specifically, the UG requires two players, the proposer and the respondent, to divide a money sum. Specifically, the proposer can decide how to divide the sum and the respondent can accept or reject the offer. If the respondent refuses the offer, no player receives money. This paradigm has proven to be useful for investigating moral decision-making because participants can judge the choices’ benefits and risks by showing a more explicit knowledge of the objective probability distribution on the possible results [8].

Furthermore, the growing interest of neurosciences and the use of neuroscientific tools for the investigation of processes underlying moral decision-making, unlike the previous self-report or evaluation studies [9], have allowed researchers to deeply observe the conscious and unconscious neurophysiological correlates of moral behavior [10]. Indeed, some studies that have recorded individuals’ electrodermal and cardiovascular activity, skin conductance response (SCR), heart rate (HR), and heart rate variability (HRV), have shown that these indexes provide information about highly positive or negative emotions experienced during moral decision-making [11,12]. Specifically, research conducted on healthy individuals showed a different activation of SCR, as a measure of emotional arousal, and HR, an index of emotional engagement, concerning fair and unfair moral decisions consequences [13]. Moreover, some studies have shown that there is a decrease in HR frequency during the experimentation of negative emotions compared to positive ones during moral decision-making [14].

Therefore, in the present study, a task, consisting of a modified UG version, was created to investigate moral decision-making in a company context. Specifically, this task proposes three different moral contexts (professional fit, company fit, and social fit) and offers: fair, unfair, and neutral. Despite the fact that company moral decision-making has recently received increasing attention, the used approaches in research have excluded the individual and situational variables underlying moral behaviors [15]. In this regard, the main aim of the present study was to investigate the different managers’ autonomic responses (SCR, HR, HRV) in response to these three moral contexts and offers type. In addition, this research aimed to investigate how utilitarianism, fairness and unfairness perception and choices prosocial and social implications influence decision-making in a company context. In order to investigate how these factors can influence moral decision-making, individuals’ autonomic responses have been recorded during different moral decisions contexts. Autonomic activity, indeed, can provide information on individuals’ levels of personal involvement and emotional engagement experienced during moral decisions regarding different contexts. In particular, as demonstrated by previous studies [16,17,18], HR variations can provide information on the emotional impact and salience of moral decision context for individuals, while HRV variations can inform about individuals’ attentional and cognitive levels during decision-making processes [19,20]. Moreover, as demonstrated by other studies [14,21], SCR variations can provide information on individuals’ level of emotional arousal experienced according to moral decisions’ benefits or losses. In light of this evidence, the information on emotional, cognitive and attentional processes underlying moral decision-making provided by autonomic measures can lead the company to develop new managing and leadership models for the organizational team that allows an adequate assessment of personal and social decisions possible implications. Moreover, the investigation of processes underlying moral decision-making in company context appears to be fundamental since company’s decisions can produce positive or negative social consequences regarding the health and the well-being of consumers, employees, and organizational community, causing multiples effects on organizational culture quality [22,23]. In addition, it is useful to deeply investigate company moral decision-making, which can be considered a very complex process influenced by individual, contextual, and situational factors.

In the company context, indeed, the moral decision process foresees possible implications and risks relevant to health and at social level [24]. The relevance of moral decision-making in company has been demonstrated by some studies that have observed the differences in decisions moral intensity assessments between managers and public people, finding that managers’ assessments were more influenced by certain factors, such as social consensus, the response extent, and the action risk, compared to public people decisions, which can improve their physiological responses [25,26].

Specifically, we hypothesized to observe a different increase of HR and HRV, as a consequence of higher emotional engagement and attentional-emotional regulation, related to the three different choices conditions. Indeed, as demonstrated by previous studies [17,18], HR provides information about the emotional salience and impact of a situation for individuals. Instead, regarding HRV, some studies [19,20] have shown that this index appears to be implicated in individuals’ attentional and cognitive levels during decision-making processes. In particular, in the professional fit condition, in which the individual is more personally involved and emotionally engaged caused by personal interests, we expected to observe an HR increase compared to company and social fit. Furthermore, we expected to observe an HRV increase in company fit condition compared to professional and social fit ones, due to a greater attentional focus and cognitive control about their working environment. Finally, we expected to observe an SCR increase for fair offers compared to unfair ones in all choices conditions, due to the higher moral acceptability of this offer type. Indeed, as demonstrated by previous studies [14,21], SCR can provide information about physiological autonomic response under different emotional constraints and rewards or punishments [13].

## 2. Method 

### 2.1. Participants

18 managers (M_age_ = 43.71; SD_age_ = 11.56) coming from different companies with similar profiles (non-governmental companies) took part in the research after signing the informed consent. The following exclusion criteria were used for all participants: normal or correct-normal visual acuity and absence of neurological or psychiatric pathologies. Moreover, the following inclusion criteria were used: age between 25 and 60 years, obtained at least a high school education, and managerial position occupation.

The research was conducted according to the Helsinki Declaration and was approved by the local ethics committee of the Department of Psychology of the Catholic University of Milan. No specific sample size hypothesis was adopted. Moreover, to safeguard the integrity of the participants’ responses, the test was performed anonymously and not used for future company use. 

### 2.2. Procedure 

The subjects were seated in a room in front of a computer monitor placed at a distance of 70 cm. The experiment consisted of a task development administered through the E-Prime 2.0 software (Psychology Software Tools, Inc., Sharpsburg, Pennsylvania, USA). During the task administration, individuals’ autonomic responses were recorded via a Biofeedback system. In particular, the task required participants to make some decisions about certain hypothetical situations that were shown to them in which some offers, that they could accept or refuse, were presented. Furthermore, it was specified to the participants that the decisions concerned three different contexts. In particular, three different randomized moral decision-making choice contexts were proposed: professional fit, company fit, and social fit. Specifically, in the professional fit condition subjects were required to accept or reject the money sum subdivision (1000 euros) proposed by a colleague for a work done together (i.e., “Your boss hires you for an extra remunerated job with a bonus of €1000 together with your colleague Mary. Your boss explains to you that, when the work is finished, the job bonus must be divided in some way between you and Mary; otherwise, no one will get money. When the work is finished, you realize that you and Mary have worked fairly on the project. Mary offers you options on how to divide the sum: 60% you and 40% Mary; 50% you and 50% Mary; 40% you and 60% Mary”). Instead, in the company fit condition, they were required to accept or refuse the money sum subdivision (1000 euros) proposed by the company for the realization of some company benefits (i.e., “The company in which you work decides to give you a bonus of €1000 due to an increase in profit. The company proposes you to help the increase of company benefits using a sum of your bonus. For example, the company plans to build a residence shortly and makes proposals for the residence’s construction, including a percentage of your bonus. So, at the end of the month, you need to decide how much of your bonus to invest in your company for the construction of the residence: 50% you and 50% company residence; 40% you and 60% company residence; 60% you and 40% company residence. If you decide to reject the company’s proposals, the latter will not be able to plan the creation of the residence for the next year”). Finally, in the social fit condition, subjects were required to accept or refuse the money sum subdivision (1000 euros) proposed by the company to support a colleague’s relative with health problems financially (i.e., “Your company decides to give you a bonus equivalent to € 1000, following a profit increase. The company tells you that with a part of this bonus you can also contribute to a just social cause outside the company. Recently, one of your colleagues had to pay a lot of money for his wife’s cancer treatment and asked the company to propose, to those employees interested, to give a percentage of their bonus to go to his wife’s treatments. At the end of the month you need to decide how much of your bonus to invest for this cause: 40% you and 60% colleague’s wife; 60% you and 40% colleague’s wife; 50% you and 50% colleague’s wife. If you decide to reject the company’s proposals, your colleague’s wife cannot be treated”).

During each situation, three other randomized offers types (fair, unfair, and neutral) were proposed. Specifically, the neutral offers proposed an equal money subdivision in all the three contexts (50% respondent and 50% bidder); the fair offers proposed a favorable money subdivision for the respondent (60% respondent and 40% bidder); finally, the unfair offers proposed an unfavorable money subdivision for the respondent (40% respondent and 60% bidder). For each offer, subjects were reminded that if they refused, neither would get the money.

The trial structure was composed by the development of three blocks that proposed three different choice conditions (professional fit, company fit, and social fit). In particular, each block lasted about 15 minutes and was composed as follows: an initial blank screen presentation, the choice condition presentation (professional fit, company fit, or social fit), the scenario presentation, the first offer presentation, the blank inter-stimulus with a cross in the center of 14 second duration, the second offer presentation, a blank inter-stimulus with a cross in the center of 14 second duration, the third offer presentation, and a blank inter-stimulus with a cross in the center of 14 second duration. In particular, the block started with the presentation of the choice condition, that could be professional fit, social fit or company fit, which was randomized between participants. Then, participants were presented with a scenario that proposed a choice situation relative to that specific context. At the end of the scenario presentation, participants have to press the computer space bar to continue the task development, that proposed him to accept or reject three different offers that could be advantageous (fair) or disadvantageous (unfair) for the participant or neutral. In particular, each block (professional fit, company fit, and social fit) proposed 15 randomized scenarios, for a total presentation of 45 scenarios during the entire task development. The 15 scenarios of each condition proposed choice situations similar to each other, with small variations to avoid boring the participants during the task development. Moreover, for each scenario of each condition, three different offers (fair, unfair, and neutral) were proposed, for a total of 135 offers. In particular, 45 fair, 45 unfair, and 45 neutral offers were presented. Participants could accept or reject the proposed offer by pressing the “1” and “0” keys on the computer keyboard and they had no time limit. Before the beginning of the task, a 15 minutes familiarization task with the same structure was presented to participants. Specifically, the familiarization task proposed participants three scenarios with three offers to accept or reject for each condition (professional fit, company fit, and social fit).

### 2.3. Autonomic Measures Recording 

The autonomic activity was recorded using Expert2000 portable Biofeedback system with a MULTI radio module (Schuhfried GmbH, Mödling, Austria).

The multipurpose integrated sensor was placed in correspondence to the distal phalanx of the non-dominant hand second finger. SCR and HR data were sampled at 40 Hz. The cardiovascular activity was collected via photoplethysmography and HR data were computed starting from peripheral blood volume measures. SCR data were directly computed by the recording software by applying a 0.05 Hz high-pass filter. An online notch filter (50 Hz) was used to minimize electrical noise. Recordings were preceded by a 120 seconds baseline recording. Inter-beat interval (IBI) metrics were computed starting from raw HR data. Finally, we extracted the mean HR and the IBI standard deviation for each experimental condition. The computation of the standard deviation of IBI mirrors high-frequency components of HRV information corresponding to the vagal influence on cardiovascular activity [27].

### 2.4. Autonomic Data Analyses 

For statistical analysis, a repeated measure ANOVA was applied to each index (SCR, HR, HRV) with Condition (Professional fit; Company fit; Social fit) and Type (fair, unfair, and neutral offers) as repeated factors. For all the ANOVA tests, the freedom degrees were corrected using Greenhouse-Geisser epsilon where appropriate, with the level of significance set at 0.05. Additionally, post-hoc comparisons were applied to the data. A Bonferroni test was applied for multiple comparisons. 

## 3. Results 

**Figure 1:**
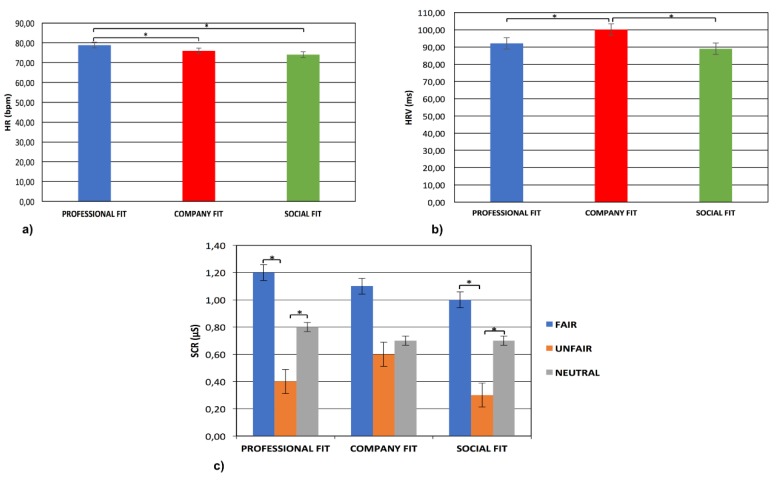
(**a**) The figure shows a heart rate (HR) increase in professional fit condition compared to others. Bars represent ± 1SE. Stars mark statistically significant (*p* < 0.05) pairwise comparisons. (**b**) The figure shows a heart rate variability (HRV) increase in company fit condition compared to others. Bars represent ± 1SE. Stars mark statistically significant (*p* < 0.05) pairwise comparisons. (**c**) The figure shows a skin conductance response (SCR) increase for fair and neutral offers compared to unfair ones in professional and social fit conditions. Bars represent ± 1SE. Stars mark statistically significant (*p* < 0.05) pairwise comparisons.

For HR, the Condition effect was significant (F(2,17) = 8.90, *p* < 0.01, η^2^ = 0.29), with an HR increase in professional fit condition compared to company fit (F(1,17) = 8.12, *p* < 0.01, η^2^ = 0.28) and social fit ones (F(2,17) = 7.89, *p* < 0.01, η^2^ = 0.27) (Figure 1a).

For HRV, the Condition effect was significant (F(2,17) = 9.33, *p* < 0.01, η^2^ = 0.32), with an HRV increase in company fit condition compared to professional (F(1,17) = 9.56, *p* < 0.01, η^2^ = 0.31) and social fit ones (F(1,17) = 9.21, *p* < 0.01, η^2^ = 0.31) (Figure 1b).

Finally, for SCR, the Condition × Type interaction effect was significant (F(4,32) = 10.77, *p* < 0.01, η^2^ = 0.33). Specifically, as revealed by post-hoc comparisons, there was an SCR increase for fair offers compared to unfair ones in professional fit (F(2,17) = 8.45, *p* < 0.01, η^2^ = 0.29) and social fit (F(2,17) = 8.10, *p* < 0.01, η^2^ = 0.29) conditions. Moreover, there was an SCR increase for neutral offers compared to unfair in professional (F(1,17) = 9.06, *p* < 0.01, η^2^ = 0.31) and social fit conditions (F(2,17) = 8.96, *p* < 0.01, η^2^ = 0.29) (Figure 1c).

## 4. Discussion 

The present study aimed to investigate possible differences in individuals’ autonomic responses (SCR, HR, and HRV) concerning different decision-making conditions and offers within a company context. Specifically, the proposal of different moral choices contexts and offers has allowed investigating individual, situational and contextual factors that can influence moral decision-making. In particular, the professional and company fit choice conditions have allowed investigating the influence of utilitarian assessment, involving attentional and cognitive mechanisms used for the assessment of the primary and secondary decisions benefits. Finally, the social fit choice condition has allowed investigating the influence of emotions in moral decision-making. This task, therefore, has proven to be useful to investigate how individual factors, such as personal interests and fairness, contextual factors, regarding the decision context, and situational factors, related to decision personal and social implications, can influence moral decision-making.

The analyses conducted allowed us to report the following main results. Firstly, considering HR, a significant effect was observed for condition with an HR increase in professional fit condition compared to others. This result may be due to a greater individuals’ emotional engagement for this moral situation in which individuals’ choices are more focused on personal interests regarding a money subdivision proposed by a colleague for a work done together. Indeed, as shown by some studies [16,17,18], an HR increase appears to be correlated to a higher emotional engagement that is experienced when individual perceives a situation as emotionally salient and relevant and in general more favorable for himself. Furthermore, this result is in line with some studies that have observed an HR increase during utilitarian choices due to higher impact of these choices for people [14,28].

Secondly, considering HRV, a significant increasing effect was observed for company fit condition compared to others. In line with previous research, it was shown that an HRV increase could be linked to be more focused on individuals’ attention and cognitive engagement [19,20].

Furthermore, considering HRV as an index that provides information on attentional-emotional and cognitive processes regulation [29], the increase of HRV appears to be related to a greater assessment of moral decisions possible implications [14]. In light of this previous evidence, the greater HRV activation in the company fit condition could be explained because it proposes to offer a money sum for the introduction of some company benefits within the working environment. The increase in focused attention in this situation compared to others, therefore, could be because individuals occupying a managerial position are more focused on the impact consequences of new opportunities (such a social service for the company) that could be introduced into their working environment.

Finally, with regards to SCR, an SCR increase was observed for both fair and neutral offers compared to unfair ones in professional and social fit conditions. This result may be due to the fact that, firstly, fair offers trigger more positive subjects’ response in terms of emotional arousal when subjects perceive the moral acceptability of the offer itself. Indeed, as shown by some studies that considered the SCR as a reliable measure to assess physiological autonomic response under different emotional constraints such as moral decision-making [14,21], SCR may be modulated by favorable emotional and positive social condition. More specifically, it has been observed that higher SCR is produced mostly during situations that produce a reward or punishment [13]. In this regard, an SCR increase in fair offers compared to unfair ones could be due because the former activates individuals’ brain reward circuit [30] increasing the physiological arousal associated to positive emotional responses. The increased SCR value for neutral offers compared to unfair ones, specifically for professional and social fit condition, may further support this explanation, based on subjective perception of equity as a positive (although not so much personally favorable as in a fair condition) in situations where the subjective or the social impact of the moral decision-making is more relevant. This result also highlights how in the company fit condition there is no significant difference between fair and neutral offers compared to unfair ones. This result could be due to the fact that the company fit condition more activates individuals’ attentional and cognitive mechanisms implicated in a utilitarian evaluation regarding the possible interests and gains associated to a moral decision. On the contrary, the professional and social fit conditions caused a higher activation of individuals’ emotional responses, which influenced moral decision-making.

## 5. Conclusions

To conclude, the present study underlined the importance of understanding moral decision-making within a company as individuals’ actions that can have social consequences at different individual or social levels. Furthermore, the present study shows that moral decision-making appears to be influenced not only by individual factors, but by contextual and situational factors that must also be considered. This evidence was supported by the fact that in professional and company fit condition, in which personal interests are more involved, individuals’ choices appear to be influenced by a careful evaluation based on individuals’ personal interests and possible gains, while, in the social fit condition, the evaluation of personal interest becomes secondary in decision-making. Moreover, individuals’ moral decision-making appears to also be influenced by offers of moral acceptability and fairness and unfairness perception, which activate individuals’ brain reward or punishment mechanisms [13]. The fairness and unfairness perception, indeed, appears to be a relevant factor in moral decision-making, which mainly concerns situations in which individual choices can have consequences on a personal and social level.

The investigation of the factors that influence moral decision-making allows, therefore, the formation of new managing and leadership models that can lead the company to help its working team to carefully evaluate the benefits, losses, and personal and social implications of a moral decision.

Despite the innovativeness of the paradigm used for the investigation of moral decision-making, the present study has some limitations. The first concerns the lack of adjunctive self-report measure able to better describe the subjective perception of different decisional conditions. The second limitation is regarding the fact that to opportunely differentiate and create quite specific moral choices conditions, the scenario should be changed across the conditions. The third limitation is related to the small sample size. Furthermore, in future studies, we could think of observing not only autonomic responses, but also central neurophysiological correlates related moral decision-making in different situations and offers. Moreover, in future studies, we could plan to increase the experimental sample.
